# Bone remodeling: A tissue-level process emerging from cell-level molecular algorithms

**DOI:** 10.1371/journal.pone.0204171

**Published:** 2018-09-19

**Authors:** Clemente F. Arias, Miguel A. Herrero, Luis F. Echeverri, Gerardo E. Oleaga, José M. López

**Affiliations:** 1 Grupo Interdisciplinar de Sistemas Complejos (GISC), Universidad Complutense, 28040 Madrid, Spain; 2 Departamento de Análisis Matemático y Matemática Aplicada, Facultad de Matemáticas, Universidad Complutense, 28040 Madrid, Spain; 3 Instituto de Matemáticas, Facultad de Ciencias Exactas y Naturales, Universidad de Antioquia, 53108 Medellín, Colombia; 4 Instituto de Matemática Interdisciplinar, Facultad de Matemáticas, Universidad Complutense, 28040 Madrid, Spain; 5 Departamento de Morfología y Biología Celular, Universidad de Oviedo, 33006 Oviedo, Asturias, Spain; Universite de Nantes, FRANCE

## Abstract

The human skeleton undergoes constant remodeling throughout the lifetime. Processes occurring on microscopic and molecular scales degrade bone and replace it with new, fully functional tissue. Multiple bone remodeling events occur simultaneously, continuously and independently throughout the body, so that the entire skeleton is completely renewed about every ten years.Bone remodeling is performed by groups of cells called Bone Multicellular Units (BMU). BMUs consist of different cell types, some specialized in the resorption of old bone, others encharged with producing new bone to replace the former. These processes are tightly regulated so that the amount of new bone produced is in perfect equilibrium with that of old bone removed, thus maintaining bone microscopic structure.To date, many regulatory molecules involved in bone remodeling have been identified, but the precise mechanism of BMU operation remains to be fully elucidated. Given the complexity of the signaling pathways already known, one may question whether such complexity is an inherent requirement of the process or whether some subset of the multiple constituents could fulfill the essential role, leaving functional redundancy to serve an alternative safety role. We propose in this work a minimal model of BMU function that involves a limited number of signals able to account for fully functional BMU operation. Our main assumptions were i) at any given time, any cell within a BMU can select only one among a limited choice of decisions, i.e. divide, die, migrate or differentiate, ii) this decision is irreversibly determined by depletion of an appropriate internal inhibitor and iii) the dynamics of any such inhibitor are coupled to that of specific external mediators, such as hormones, cytokines, growth factors. It was thus shown that efficient BMU operation manifests as an emergent process, which results from the individual and collective decisions taken by cells within the BMU unit in the absence of any external planning.

## Introduction

The human skeleton is a complex structure made up of 206 bones, which constitute a rigid, supportive framework for the body. It acts as a shield to protect internal organs and plays a crucial role in locomotion by anchoring the force arising from muscle contraction. In spite of its inert appearance, bone is an extremely dynamic tissue that is continuously being remodeled to adapt to changing mechanical demands. Such remodeling, which is carried out on a microscopic scale, consists in the removal of low-performing bone and its replacement by new, fully functional bone. This task is fulfilled by suitable agents designed for that purpose, as described below.

Bone tissue is formed from a mineralized matrix that has been hardened to provide a supporting function. There are three key cell types that are responsible for matrix production, maintenance and remodeling: viz. osteoclasts, osteoblasts and osteocytes which perform different homeostatic roles [1–3]. Osteoclasts, recruited when needed from their cell precursors, are in charge of degrading dysfunctional bone, whereas the biosynthesis of new bone to replace the former is carried out by osteoblasts. Osteocytes, the most abundant bone cells, form a three-dimensional interconnected network throughout the osseous tissue. They act as mechanosensors that monitor mechanical stress within bone tissues, and react to changes in both the amount and the direction of loading applied on bones.

A key event that triggers bone remodeling is osteocyte cell death (apoptosis) which occurs over comparatively short time scales at focal areas of bone microdamage and results, for instance, from unusual mechanical loads or normal daily activity. In this condition, it is noteworthy that the relationship between osteocyte apoptosis and applied load is known to be U-shaped. This means that mechanical stresses within a normal physiological range prevent apoptosis, whereas those above or below this range induce it [4–6]. In traumatic bone fractures, a considerable number of osteocytes are eliminated and alert signals are produced that recruit immune cells to result in an inflammatory response. In such cases, an alternative mechanism of bone formation is triggered to implicate other cell types [7]. We shall not deal with this case here, as we are principally concerned with homeostatic bone remodeling on smaller cellular and time scales. The manner in which this process occurs is described below.

**Fig 1:**
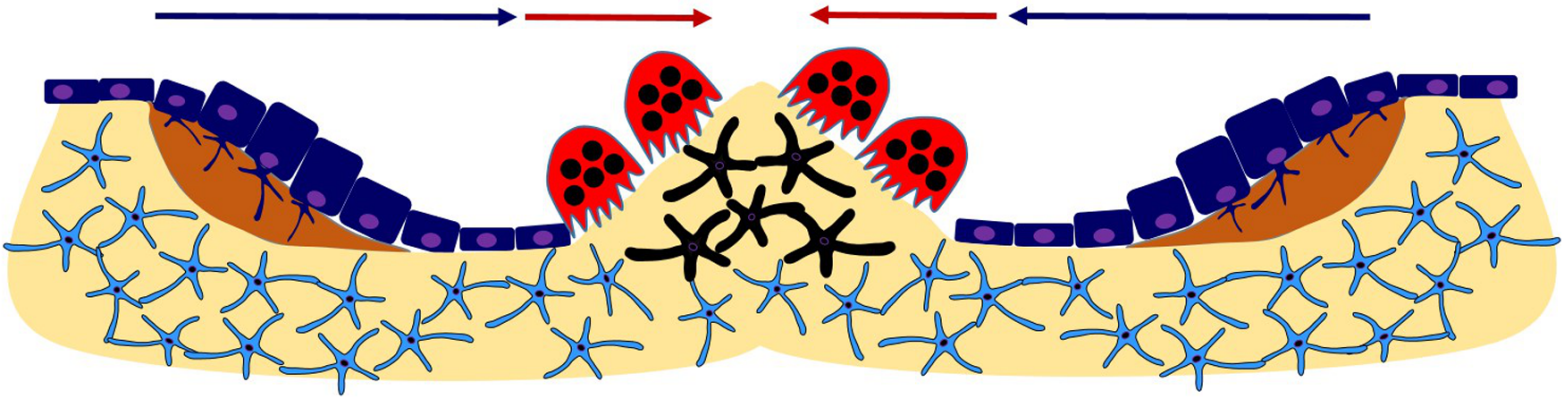
A sketch of BMU operation after a group of osteocytes has undergone apoptosis near bone surface. Bone resorption (red arrows) and bone formation (blue arrows) are performed in this order. Bone remodeling is initiated when osteoclast precursor cells are recruited to the altered bone surface (black stellate cells) and fuse to form mature, bone resorbing osteoclasts (red cells) that attach to the surface. Mature osteoclasts degrade the mineralized matrix (light yellow) and produce resorption pits also called resorption bays or Howship’s lacunae. Once osteoclasts have degraded the target area, they undergo apoptosis, and osteoblasts (dark blue cells) situated behind them first secrete osteoid matrix (dark yellow) and subsequently differentiate into mature osteocytes (light blue stellate cells).

Following osteocyte apoptosis in a microscopic region approximately 400 microns wide, termed Bone Remodeling Compartment (BRC), organic teams called Bone Multicellular Units (BMU) are recruited locally [8, 9]. Each BMU consists of several morphologically and functionally different cell types, mainly osteocytes, osteoblasts and osteoclasts, that act in coordination on the BRC to replace old bone by new one [10, 11]. Not all cell types required are initially in place. In fact, prior to remodeling, the normal presence of osteocytes produces an inhibitory effect that keeps osteoblasts deactivated and reduces osteoclast precursors, which would otherwise give rise to functionally active osteoclasts. However, osteocyte apoptosis in the BRC results in a decrease of such inhibitory action, with various consequences. To begin with, the disinhibition leads to osteoblast activation and to the recruitment of osteoclast precursors derived from the bone marrow. Such precursors subsequently differentiate into mature osteoclasts, which initiate the erosion (resorption) of adjacent bone, leading to the appearance of the so-called cutting cones [12]. In a later stage, activated osteoblasts advance in the wake of bone-destroying cutting cones to replenish the cavity left behind by the latter, by secreting an osteoid matrix, a precursor to new bone. Some of the active osteoblasts become trapped in the matrix that they secrete and eventually differentiate into osteocytes [13], which in turn would be instrumental in the subsequent mineralization of the matrix surrounding them, thus concluding a local bone remodeling event. The whole process is summarized in Fig 1.

It should be stressed that the entire process described in Fig 1 is exquisitely regulated. It only takes place where needed and the amount of new bone generated precisely balances that of old bone destroyed, thus warranting that no net changes in bone mass nor mechanical stress remain after each remodeling cycle. Any imbalance between bone resorption and bone formation could lead to malfunction in bone remodeling which may result in pathological disorders like osteoporosis, renal osteodystrophy, Paget’s disease, osteopetrosis or rickets.

To date, and in spite of substantial progress achieved during recent decades, the mechanisms of cell signaling that regulate BMU operation remain only partially understood. It is known that such regulation is of a local nature, since remodeling occurs simultaneously and independently at different sites, and is performed in a systematic manner so that the whole adult skeleton is continuously renewed [14]. The local nature of that process suggests that it is likely to be a consequence of individual cell decisions that must somehow be coordinated at a population level. For instance, osteoblasts should not start secreting osteoid matrix before osteoclast-mediated destruction is finished. This immediately raises the questions of how such coordination can be achieved, and what internal circuitry within a BMU keeps such units operative when remodeling is needed, and what shuts it off immediately afterwards. We shall address these issues in the following sections.

Specifically, in this work we propose and analyse a simple, space-dependent model that is able to reproduce BMU operation and requires only of a small number of signaling cues. In our model, BMU function is shown to result from a limited number of individual decisions (divide, die, migrate or differentiate) taken by any of the cells involved due to the coupled dynamics of external cues and internal decision inhibitors. Simplicity is a key goal here. We are in fact interested in describing a minimal software sufficient to ensure BMU operation and involving generic molecules able to elicit actions that have already been experimentally observed. We therefore do not aim to provide a comprehensive model of bone remodeling requiring all or most of the implicated pathways. Rather, the aim was to identify the minimal circuitry needed to keep a BMU operative. We believe that this may be a useful complement to previous work (see for instance [1, 15–19] and references therein) where attention has instead been paid to building comprehensive mathematical models. We hope that a combined use of both approaches (comprehensive and minimality-driven) could shed further light on our understanding of bone remodeling and on the regulatory controls involved in this process, when specific action(s) may be needed.

## Models

### The role of molecular signaling in bone remodeling

To date, a considerable number of signals involved in BMU operation have been identified in the literature [10, 14, 20, 21]. Osteocytes are key players in the regulation of bone. They produce many signaling molecules including sclerostin, a secreted glycoprotein that inhibits both osteoblast activation and osteoclast recruitment [10, 22]. This inhibitory effect prevents the activation of BMUs in regions where bone remodeling is not needed. In a given area, the inhibitory effect of sclerostin is significantly depleted only if an appropriate number of osteocytes undergo apoptosis due, for instance, to microfractures. Decreased sclerostin levels invoke the activation of a BMU and the subsequent initiation of a bone remodeling event.

On the other hand, a set of signals known under the generic name of Bone Morphogenetic Proteins (BMPs) have also been shown to play a key role during the early stages of bone remodeling. Some members of this family, such as TGF-*β* and TGF-*α*, are known to foster the differentiation of mesenchymal stem cells to osteoblasts [23, 24]. TGF-*β* can also induce migration of osteoblasts to the sites of bone formation during remodeling [25, 26] and inhibit osteoblast apoptosis [5]. TGF-*β* is mainly produced by osteocytes [22, 27], but it is also present in the bone matrix [23] and in platelets [28]. This latter factor, together with other signals such as HMGB-1 [29] provides a link with inflammatory processes occurring in early stages of large fractures repair. Cytokines such as IGF-1, released from bone matrix, seem to play a similar role in activating osteoblast differentiation [30] and are necessary for their survival in vitro [31].

The next stage in the process of bone remodeling consists in the recruitment of osteoclasts. The main signals involved in this step are M-CSF and RANKL [21, 32, 33]. They promote the differentiation of osteoclast precursors and the survival of activated osteoclasts. Since RANKL is mostly produced by activated osteoblasts [10, 32], osteoclasts will only be recruited to sites were bone remodeling has already been triggered. Sclerostin both inhibits osteoblast activation and induces apoptosis of active osteoclasts [10, 22]. It may thus act as a signal to stop bone resorption when the cutting edge has reached the required depth in each remodeling event. Moreover, osteoid matrix production by active osteoblasts, as well as differentiation of osteoblasts into osteocytes seem to be cell-density dependent [34], and have been suggested to be mediated by connexin, a molecule that circulates through gap junction communications between osteoblasts, as well as by sclerostin [20, 35]. Finally, various chemoattractants/chemorepulsors that drive osteoclasts away from the region where their precursors are recruited have been described in the literature (see for instance [36]). We shall use one such generic signal as part of our algorithm below.

Concerning signaling effects, one must bear in mind that: i) different signals can have redundant effects; for example both TGF-*β* and IGF-1 induce osteoblast differentiation [37], ii) a given signal can have different effects on different cell types. For instance TGF-*β*, FGF and PDGF activate osteoblast and inhibit osteoclast action [28] whereas G-CSF is known to induce apoptosis and inhibit differentiation in osteoblasts [33], and iii) signals are not always provided by chemicals. In this context we have already remarked that osteocytes act as sensors responsive to changes in mechanical stress of bone [38, 39].

The preceding list of molecular mediators and their effects on BMU cell types is presented in Table 1 below as a concise summary of a more complex scenario. A limited knowledge of the identity of such mediators or describing their effects in qualitative terms is not enough to explain BMU operation during bone remodeling. In order to understand how a coherent collective plan of action emerges at a multicellular scale, quantitative aspects of the process need to be taken into account. Indeed, for any given set of signals involved, the amount of bone to be resorbed and produced in different remodeling events can be highly variable [10]. This implies in turn that the number and activity of cells recruited in a BMU should change to suit the needs of each particular remodeling process [27, 28, 40, 41].

**Table 1:** A brief summary of the main signals involved in BMU operation as described in the literature (references are provided in the text). Abbreviations for cell types are as follows. OCY: Osteocytes, aOCY: apoptotic osteocytes, OBL: osteoblasts, OBA: activated osteoblasts, OCL: osteoclasts. Each entry in a row contains (from left to right) the name of one (or several) signals, its main source, and its effect on the cell types sequentially listed. For instance, the third line in the table asserts that sclerostin is produced by Osteocytes, prevents Osteoclasts recruitment and inhibits proliferation and differentiation and induces apoptosis in both Osteoblasts and activated Osteoblasts.

Signal	Source	Target cells		
		OCL	OBL	OBA
HMGB-1	aOCY	Recruitment Chemotaxis	Recruitment Chemotaxis	Chemotaxis
RANK, OPG M-CSF	OBA aOCY	Recruitment Activation Survival	-	-
Sclerostin	OCY	No recruitment	No proliferation No differentiation Apoptosis	No proliferation No differentiation Apoptosis
TGF-*β* BMPs PDGF IGF FGF	OCY OBL OBA	Deactivation	Chemotaxis Recruitment Differentiation Proliferation Survival	Chemotaxis Differentiation Proliferation Survival

### Signal integration by individual cells. Inhibitory proteins

We now formulate our basic modeling assumptions, which can be summarized as follows. Cells within a BMU integrate signals from their immediate surroundings and the outcome determines a very limited set of actions, namely differentiation, cell division, migration and/or apoptosis. In addition, we propose that inhibitory proteins that impede the progression of these actions within each cell type, mediate cell decisions in any bone-remodeling event.

**Fig 2:**
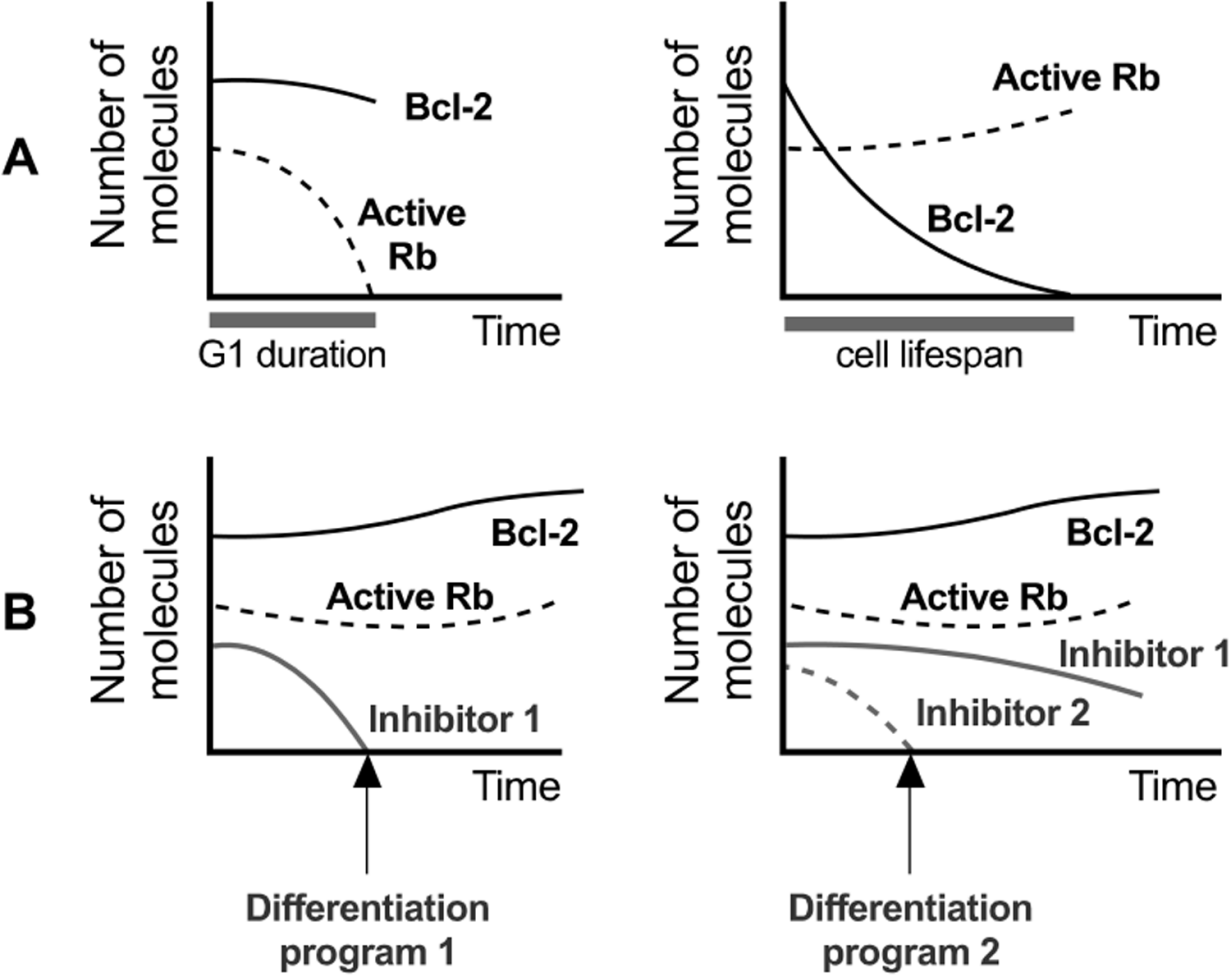
A mechanism of cell fate determination. **A)** Inhibitory proteins Rb and Bcl-2 provide a fate decision mechanism in several cell types. The first of these molecules to reach its critical threshold determines if the cell will divide or undergo apoptosis. For convenience all inhibitory thresholds are set equal to zero. **B)** The presence of inhibitors blocking alternative differentiation programs allows an increase in the complexity of this cell fate-decision mechanism. **B Left)** If differentiation is assumed to be blocked by Inhibitor 1, and this inhibitor vanishes before Rb and Bcl-2 do, then the cell will not divide or die, but will instead undergo the differentiation program 1. **B Right)** Two alternative differentiation programs can be controlled by two different inhibitors (1 and 2). If one of them (inhibitor 2 in this case) disappears faster than the remaining molecules, the corresponding differentiation program is selected.

To clarify this last assumption, it is worth recalling the well-known behavior of two inhibitory proteins, Rb and Bcl-2. The retinoblastoma protein (Rb) arrests progression into the cell cycle, whereas the B-cell lymphome-2 protein (Bcl-2) precludes the initiation of the apoptosis program in most cell types, including osteocytes and other stromal cells [38]. More precisely, Rb binds to transcription factors of the E2F family, preventing the progression of the cell cycle to the synthesis stage [42]. When the amount of active Rb falls below a critical threshold, a point of no-return is reached (the Restriction Point of the cell cycle) that irreversibly leads to cell division. Analogously, Bcl-2 precludes the release of cytochrome c through the mitochondrial outer membrane, thus avoiding the initiation of the apoptosis program. If Bcl-2 is depleted beneath a suitable level, its inhibitory action is lost and the cell is committed to death [28, 43]. Hence a competition between inhibitory molecules results in a mechanism of cell fate control: the first of these inhibitors (Rb or Bcl-2) that falls below its corresponding threshold value determines the decision of the cell between dividing or dying and, importantly, the timing of this decision (see Fig 2A). This mechanism also provides an explicit link between external signals and cell decisions, since membrane receptors are known to modulate the evolution of inhibitory molecules within the cell [43]. As a matter of fact, it has been recently shown that the interplay between receptor/signal interaction and the internal dynamics of Rb and Bcl-2 suffices to explain the onset of emergent population properties (as clonal expansion and contraction) in the case of immune response to acute infections [44], see also Fig 2.

In our case, the occurrence of inhibitory proteins controlling cellular processes during bone remodeling is well documented in the literature. For instance, the roles of Rb, Bcl-2 and the transcription factor Runx2 have been described in [45–47] respectively. Moreover, different restriction points are known to occur for the various cell commitment alternatives involved in bone remodeling. In particular, the onset of two restriction points in the differentiation program of osteoblasts (marking respectively the transition to activated osteoblast and osteocyte types) has been pointed out in [28].

The increased complexity derived from the presence of more than one cell type, together with the existence of several cell fates (division, apoptosis or alternative differentiation programs) introduces new possibilities with respect to the basic dichotomy between cell division vs. cell death [44]. However, the underlying logic can be extended to account for these new alternatives. For instance, several inhibitory molecules can simultaneously block the progression of alternative differentiation programs. In this case, the first inhibitor to reach its critical threshold will determine the fate of the cell (see Fig 2B). We propose that cell choices thus determined are mutually exclusive. This seems to be the case for BMU cells. For instance, osteoblasts that start the differentiation program or decide to secrete osteoid matrix do not complete the cell cycle, and therefore do not proliferate [28]. We also remark that this mechanism allows for one signal to trigger alternative cell decisions depending on its concentration.

Bearing in mind the multiplicity of signals collected in Table 1, as well as the redundancy often observed in their functioning, we propose that the effective operation of a BMU can be modeled by means of a reduced version of the complex signaling network previously sketched. Specifically, we propose that three cell-released signals, denoted by *R*, *S* and *T* (with analog roles to those of RANK, sclerostin and TBF-*β* respectively; see Table 1) plus one osteoclast cue (see [36]), that keeps such cells moving towards intact bone, acting on three types of internal inhibitors suffice for that purpose. The effect induced by such signals in the cell types involved is described in Table 2.

**Table 2:** Generic signals to be considered in our model and a list of the actions they induce on cell types of a BMU. Abbreviations as in Table 1.

Signal	Source	Target cells		
		OCL	OBL	OBA
R	OBA	Activation Survival	-	-
S	OCY	No recruitment	No proliferation No differentiation Apoptosis	No proliferation No differentiation Apoptosis
T	OCY OBL OBA	Deactivation	Proliferation Differentiation Survival	No proliferation No differentiation Apoptosis

### A model for BMU operation

We next describe the cellular algorithms that constitute our proposed model. For simplicity, we will consider a two-dimensional cross section of bone adjacent to a section of bone marrow. The bone section considered will be thought of as a lattice with coordinates *x*, *y*, divided in boxes of equal size. Any such box can either remain empty or be occupied by only a single cell. On such a region we define a cellular automaton (CA) to describe the dynamics of the remodeling process. To that end, we implement cellular algorithms based on the biological assumptions stated below. A first assumption is that progression within the cell cycle, apoptosis and the initiation of differentiation programs are initially blocked by specific inhibitory molecules as described above. This would allow for newly formed cells to remain in the first stage of the cell cycle (G1) before choosing a given cell fate. During this stage, membrane receptors interacting with external signals govern the dynamics of inhibitors, thus controlling the eventual decision of the cell. The effect of the complexes formed by membrane receptors and external cues in the inhibition (activation) of any cell fate choice takes place through an increase (decrease) in the amount of the inhibitory molecule controlling this choice. Cell fate is decided when the concentration of the first of the inhibitors considered attains its threshold value, which we set equal to zero for simplicity.

**Fig 3:**
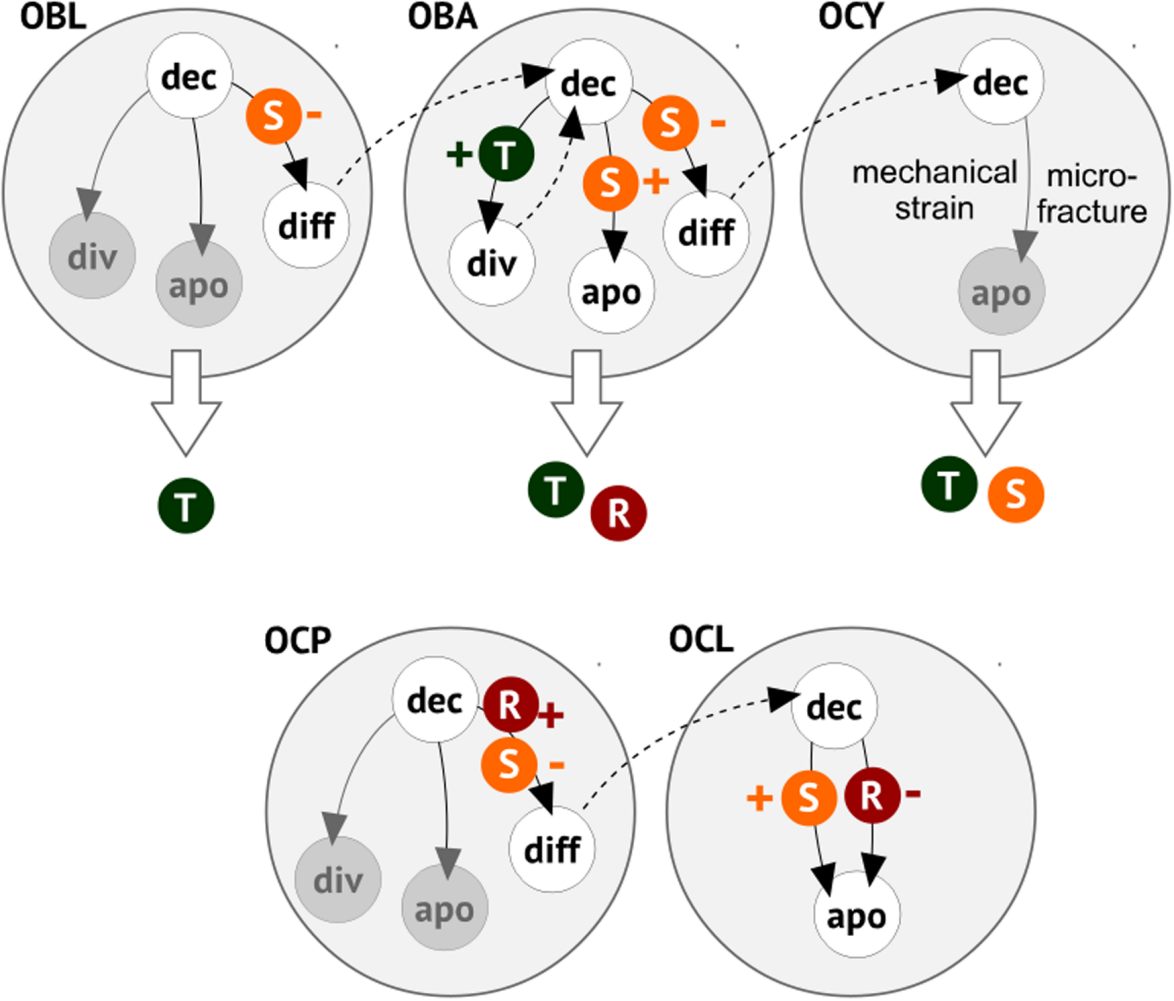
A minimal model of BMU software. Signals *R*, *S* and *T* (in small circles) are produced by the cell types listed. They may inhibit (-) or induce activation (+) on the actions considered. Activation results from double inhibition, that is by lowering the concentration of an inhibitor. Within any cell type, possible decision choices are indicated by thin arrows. Dashed arrows correspond to a starting decision stage in a newly formed cell. Extracellular release of signals by any cell type (for instance, *R* and *T* in the case of active osteoblasts) is denoted by thick, white arrows.

In our case, the inhibitor dynamics will be assumed to be of the following form:

$$\begin{array}{c} \left\{ \begin{array}{c} c^{\prime }\left(t\right)=f_{c}\left(R,S,T\right)\\ a^{\prime }\left(t\right)=f_{a}\left(R,S,T\right)\\ d^{\prime }\left(t\right)=f_{d}\left(R,S,T\right), \end{array}\right. \end{array}$$ (1)

where *c*(*t*), *a*(*t*) and *d*(*t*) respectively denote the concentrations of division, apoptosis and differentiation inhibitors at time *t* and *f*_*c*_, (here and henceforth, prime denotes time derivative) *f*_*a*_, and *f*_*d*_ are functions of three external signals *S*, *R* and *T* (see Fig 3). For simplicity, we shall assume in the sequel that such functions will be piecewise linear. We next describe the main details of the decision algorithm proposed to describe BMU operation.

#### Osteoblasts (OBL)

Osteoblasts (OBL) are initially located at the interface between bone matrix and bone marrow. In normal conditions, osteoblast homeostasis is maintained by a continuous cell turnover, involving both division and apoptosis [48]. When a remodeling process starts, OBLs can choose among three alternative programs: division, apoptosis and differentiation into active osteoblasts (OBAs). We shall assume that OBL division and apoptosis just balance each other, and therefore focus on the third choice above. Activation is known to be mainly blocked by the release of sclerostin by osteocytes [6, 10]. This will be modeled by assuming that signal *S*, produced by osteocytes, increases the amount of a differentiation inhibitor in OBLs, denoted by *d*_*B*_, according to the following dynamics:

$$\begin{array}{c} \left\{ \begin{array}{c} d_{B}^{\prime }\left(x,y;t\right)=\alpha_{B}^{S_{1}}S\left(x,y;t\right)-\alpha_{B}^{S_{2}},\;\text{if}\;d_{B}<D_{B}\\ d_{B}^{\prime }\left(x,y;t\right)=0\;\text{otherwise}, \end{array}\right. \end{array}$$ (2)

where (*x*, *y*) denotes the position of the OBL, *D*_*B*_ is the maximum amount of inhibitor that can accumulate within a OBL, *S*(*x*, *y*; *t*) is the amount of signal *S* at location (*x*, *y*) at time *t* and $\alpha_{B}^{1}$ and $\alpha_{B}^{2}$ are positive structural parameters. During a bone remodeling process, osteoblasts remain in their original positions, delimiting the BRC [11]. Accordingly, OBLs do not move in our model. We recall that differentiation of a particular OBL is blocked while *d*_*B*_ > 0, and that the process of differentiation is triggered by the condition *d*_*B*_ = 0.

#### Active osteoblasts (OBA)

Once differentiated, OBLs become active osteoblasts (OBA). OBAs have three possible choices: cell division, cell death and differentiation into osteocytes (OCY). Cell division proceeds according to the following rules:

$$\begin{array}{c} \left\{ \begin{array}{c} c_{A}^{\prime }\left(x,y;t\right)=-\alpha_{A}^{T}T\left(x,y;t\right),\;\text{if}\;\text{box}\;\text{at}\;\left(x,y\right)\;\text{is}\;\text{not}\;\text{adjacent}\;\text{to}\;\text{bone}\\ c_{A}^{\prime }\left(x,y;t\right)=0\;\text{otherwise}, \end{array}\right. \end{array}$$ (3)

where (*x*, *y*) denotes the position of the OBA, *T*(*x*, *y*; *t*) is the amount of signal *T* at location (*x*, *y*) at time *t* and $\alpha_{A}^{T}$ is a positive parameter. Cell division only occurs when the condition *c*(*A*) = 0 is verified. Notice that, in our model, the movement of active osteoblasts front is just a consequence of cell division.

When OBAs are attached to the bone surface, cell division is no longer possible for lack of space. In that case OBAs secrete osteoid matrix; this is represented by an order parameter *b*(*x*, *y*; *t*) which ranges from 0, corresponding to eroded bone, to value 1 for fully functional bone. Apoptosis, differentiation into OCY and osteoid production can take place, according to these equations:

$$\{\begin{array}{l} a_{A}^{\prime }\left(x,y;t\right)=-\alpha_{A}^{S_{1}}S\left(x,y;t\right)\\ d_{A}^{\prime }\left(x,y;t\right)=\{\begin{array}{l} \alpha_{A}^{S_{2}}S\left(x,y;t\right)-\alpha_{A}^{S_{3}}\text{,}\;\text{if}\;\text{distance}\;\text{to}\;\text{OCYs}\;\text{is}\;\text{larger}\;\text{than}\;\delta_{A}\\ 0\;\text{otherwise} \end{array}\\ b^{\prime }\left(x,y;t\right)=\{\begin{array}{l} \alpha_{A}^{O}\text{,}\;\text{if}\;0\leq b\left(x,y;t\right)<1\\ 0\;\text{otherwise} \end{array} \end{array}$$ (4)

where *S*(*x*, *y*; *t*) is the amount of signal *S* at location (*x*, *y*) at time *t*, $\alpha_{A}^{S_{i}}$ (for *i* = 1, 2, 3) are positive structural parameters, $\alpha_{A}^{O}$ the rate of bone production, and *δ*_*A*_ is a positive constant that represents a threshold in cell-to-cell contact inhibition by OCYs on OBA differentiation, resulting from gap junction communications between cells [10, 13, 49].

#### Osteoclast precursors (OCP)

OBA can recruit OCP to the bone remodeling zone by secreting signal *R*, while signal *S* inhibits OCP recruitment. For simplicity, we will not consider in the model OCP division and apoptosis, and assume instead that enough OCP are available in the bone marrow (in particular, in the locations adjacent to those occupied by OBAs). OCPs attach to the bone surface upon their recruitment by OBAs, after which they become active osteoclasts (OCL). We assume that OCP activation is blocked by inhibitor *d*_*P*_, whose dynamics is modeled as follows:

$$\begin{array}{c} d_{P}^{\prime }\left(x,y;t\right)=-\alpha_{P}^{R}R\left(x,y;t\right)+\alpha_{P}^{S}S\left(x,y;t\right) \end{array}$$ (5)

where *R*(*x*, *y*; *t*) and *S*(*x*, *y*; *t*) are respectively the amounts of signal *R* and *S* at location (*x*, *y*) at time *t* and $\alpha_{P}^{1}$ and $\alpha_{P}^{2}$ are positive parameters.

#### Osteoclasts (OCL)

Once recruited to the BRC, OCPs become active osteoclasts (OCL) and start eroding the bone matrix, thus leading to the formation of a cutting cone [36]. OCLs are assumed to be endowed with one apoptosis pathway controlled by signal *S*, produced by OCYs. This signal is instrumental in determining the depth that will be reached by the cutting cone. In particular, OCLs in locations with high concentrations of *S* will stop digging into the bone matrix and die. We model the apoptosis pathway controlled by signal *S* by assuming that cell death is blocked by inhibitor *a*_*C*_, whose dynamics are given by the following equations:

$$\begin{array}{c} \left\{ \begin{array}{c} a_{C}^{\prime }\left(x,y;t\right)=-\alpha_{C}^{S_{1}}\;\text{if}\;S\left(x,y;t\right)>\alpha_{C}^{S_{2}}\\ a_{C}^{\prime }\left(x,y;t\right)=0\;\text{otherwise}, \end{array}\right. \end{array}$$ (6)

where *S*(*x*, *y*; *t*) is the amount of signal *S* at location (*x*, *y*) at time *t* and $\alpha_{C}^{S_{1}}$ and $\alpha_{C}^{S_{2}}$ are positive parameters.

We next describe the movement of osteoclasts during a bone remodeling event. We will assume that osteoclasts remove bone at a rate that depends on the amount of signal *S* and move to an adjacent box, along the normal to the ossification front, once they have removed bone in their current position. Bone resorption will be modeled by means of the following equation:

$$\begin{array}{c} \left\{ \begin{array}{c} b^{\prime }\left(x,y;t\right)=-\alpha_{C}^{S_{3}}\left(1-\frac{S(x,y;t)}{\alpha_{C}^{S_{4}}}\right)\;\text{if}\;S\left(x,y;t\right)\leq \alpha_{C}^{S_{4}}\\ b^{\prime }\left(x,y;t\right)=0\;\text{otherwise}, \end{array}\right. \end{array}$$ (7)

where *b* measures the presence of bone, so that *b* = 1 corresponds to functional bone, and *b* = 0 to degraded bone. *S*(*x*, *y*; *t*) is the amount of signal *S* at location (*x*, *y*) at time *t* and $\alpha_{C}^{S_{3}}$ and $\alpha_{C}^{S_{4}}$ are positive parameters.

#### Signal diffusion and decay

**Fig 4:**
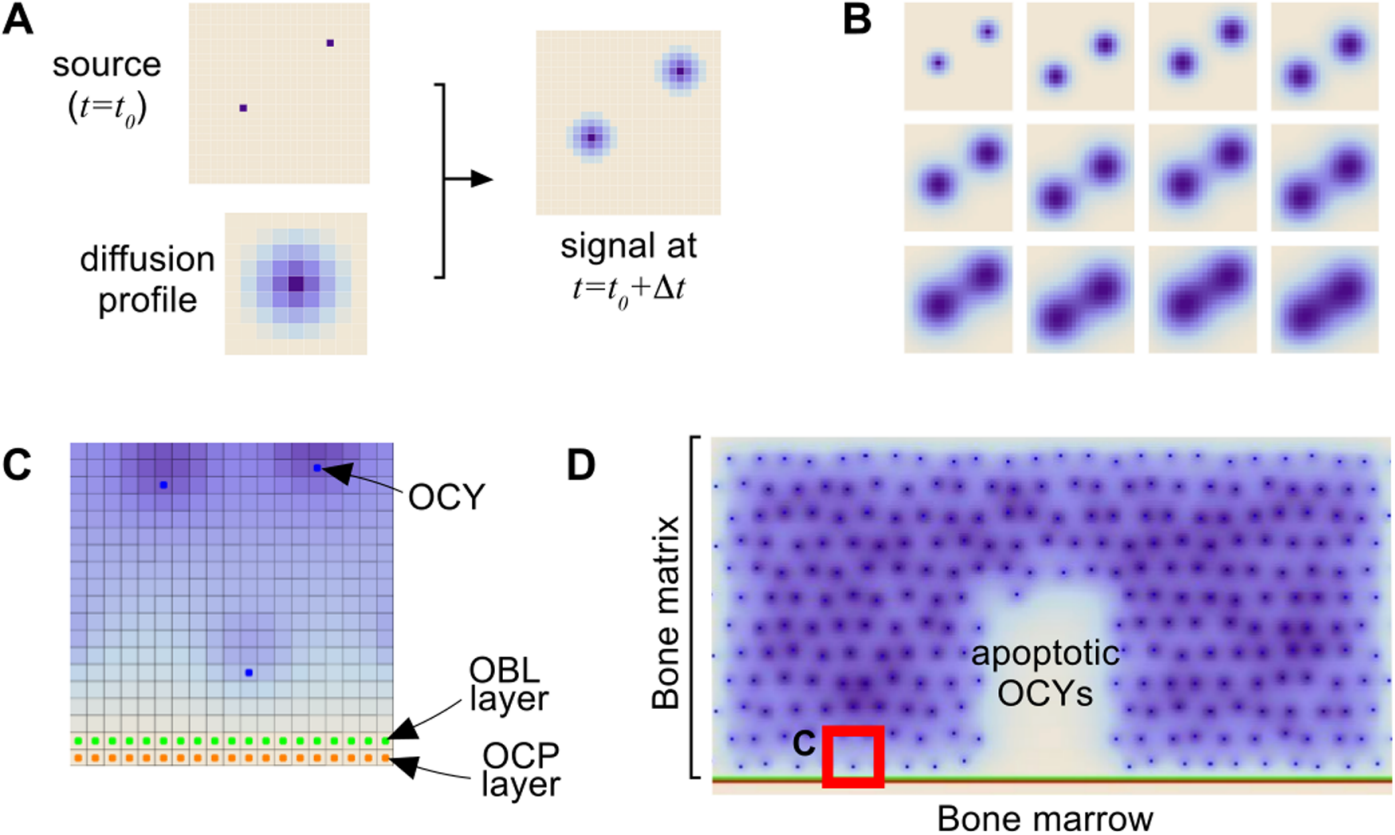
Spatial aspects of the model. **A)** Signal diffusion takes place at a different scale than cell decisions. Let Δ*t* be the characteristic time step for cell processes. In order to model how signals spread out, we calculate the diffusion profile created by the signal release from each cell during this time interval. Diffusion can then be modeled at this characteristic time by applying the corresponding profile to any source of signals in the CA. **B)** Snapshots of a signal diffusion from the source shown in A. Time increases from left to right and from top to bottom. **C)** Spatial arrangement of the main elements of the model. Bone matrix is locally represented by means of a lattice consisting of equal-size boxes, each able to accommodate one cell. The lower boundary of the box considered in C) is represented as lined with an osteoblast layer, lying upon a layer of osteoclast precursors. **D)** Initial configuration of the model. At selected sites within the bone matrix, osteocytes (blue dots) may undergo apoptosis, thus triggering BMU operation in the corresponding region (blank). The lattice element considered in C) is represented here for comparison purposes (red square in the lower left corner). Shades of blue represent different concentrations of signals in each box.

The dynamics of signals *R*, *S* and *T* in our cellular automaton (CA) model is now described. At any given time, they are produced by the corresponding cell types at constant rates. Signals are also assumed to undergo Arrhenius-type decay (meaning that their decay rate is proportional to their concentration). Signal transport through the bone matrix is conveniently represented as a diffusion process. We assume that such diffusion occurs faster than internal cell processes. In order to account for these two time scales simultaneously, we model diffusion by calculating, at each box and each iteration step of the model, a weighted average of the amount of signals in neighboring positions (see Fig 4A). Cells decisions are made upon comparing the amount of different signals in their adjacent boxes.

#### BMU initialization

As a starting point we consider a population of osteocytes (OCY), regularly distributed within the bone matrix, and a layer of osteoblasts lining the bone section (see Fig 4C). Simulations of the model are started after apoptosis of a group of osteocytes has occurred (see Fig 4D).

## Results

In this Section we present some results obtained upon simulation of the model whose elements have been described in previous Sections. To keep the flow of the main arguments here, we confine some technical details to the Supporting Information at the end of the paper. Specifically, the reader will find there a flow chart describing the order in which the corresponding algorithms are applied, as well as a list of the parameters used in the simulations whose results are shown below.

### The BMU software proposed successfully recapitulates bone remodeling

**Fig 5:**
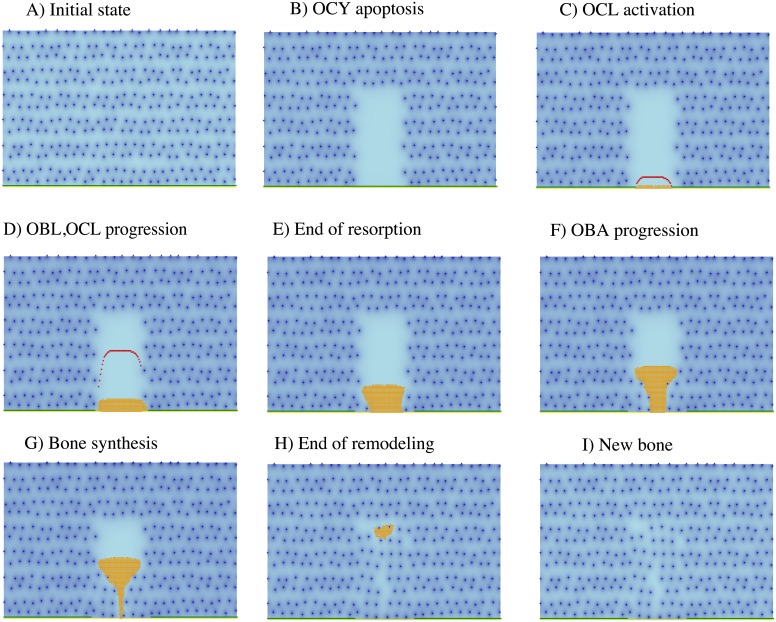
Snapshots corresponding to sequential stages in a bone remodeling event. **A**) A planar bone region is considered where active osteocytes (OCY, blue dots) are regularly distributed. Osteoblasts (OBL) are located at the lower side, at the interface between bone and bone marrow. **B)** OCY apoptosis is induced in a subset (in light blue) of the previous region, leading to a drop in OCY inhibitory action at that site. **C)** Insufficient osteocyte inhibition results in OBL activation, and the recruitment of osteoclast precursors which differentiate into active osteoclasts, OCL (in red). **D)** OCL form cutting cones that move into the apoptotic region destroying old bone (bone resorption). **E)** OCL-driven resorption proceeds until the inhibitory effect of the remaining osteocytes precludes further OCL progression. **F)** Activated osteoblasts (OBA, in orange) move in the wake of OCL. **G)** Upon successive rounds of cell division, an increasingly larger region of OBA fills the region previously occupied by decaying bone, expanding behind the OCL-lined cutting cone boundary where they secrete osteoid matrix. Some OBA subsequently differentiate into OCY (blue dots). **H)** When remodeling is finished, a new distribution of osteocytes is achieved in the remodeled region, and **I)** new bone is eventually formed. At this point, the model reaches a new equilibrium state. This allows for subsequent processes of bone remodeling to take place in the event of osteocyte apoptosis in the same bone region.

Upon induction of osteocyte death in a region, the proposed cell algorithms provide a mechanism of bone remodeling which sequentially reproduces the main phases of the actual process. See Fig 5 and its caption for more details. A video corresponding to this simulation is provided with the Supporting Information.

We point out that simulation of our model reveals that both the initial state of fully homeostatic bone, and the final stage after bone remodeling, remain stationary in time. In other words, running the model on such configurations does not result in any noticeable change in such states.

### BMU algorithm is robust to variations in the size of the BRC region

**Fig 6:**
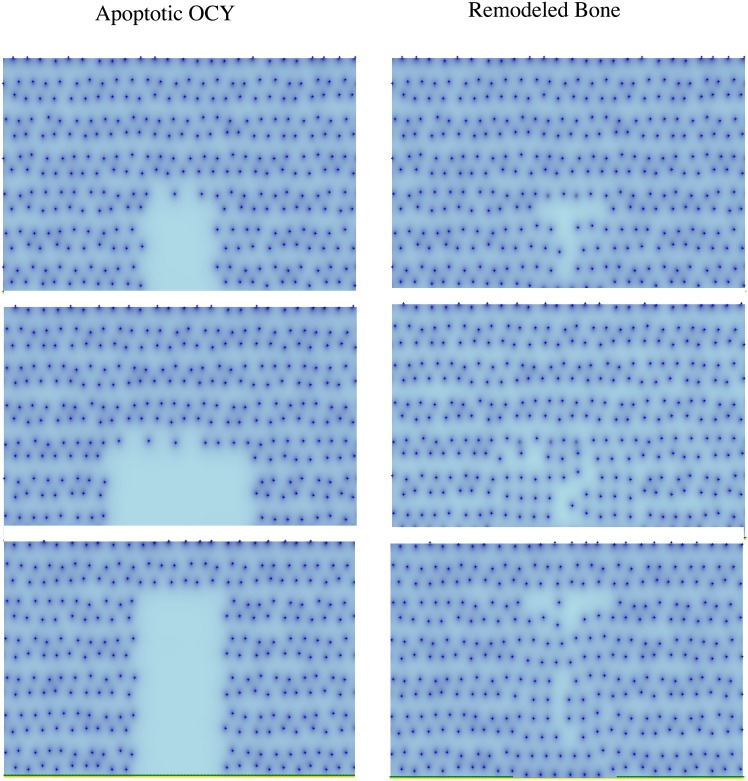
Simulation of three different scenarios of OCY apoptosis. Images correspond to results obtained in each case after simulation of the model from OCY apoptosis until the end of the remodeling process. The shape and size of the resulting BRC is determined in each case by the region where OCY apoptosis has occurred.

The model proposed can be run when OCY apoptosis is assumed to occur in regions of different sizes. In each case, the area of the corresponding Bone Remodeling Compartment (BRC) is shown to depend on the extent of the induced OCY apoptosis. See Fig 6.

### Osteoclast lifespan depends on the depth of the BRC

**Fig 7:**
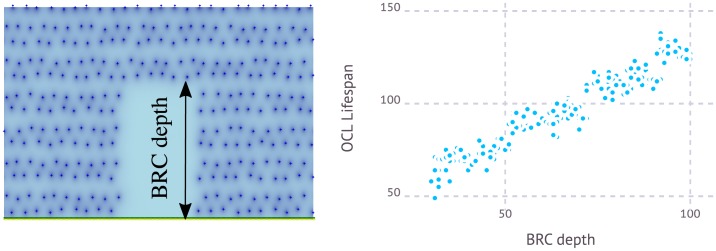
Dependence of OCL lifespan on BRC size. **Left)** A region where bone resorption has occurred following OCY apoptosis is depicted. BCR depth is defined therein as the longest distance within that region measured perpendicular to its lower side. **Right)** A plot of OCL lifespan during bone remodeling events in terms of BRC depth obtained after simulations of the model (each dot corresponds to a simulation). Note that OCL live longer when BRC is larger.

A prediction of the model is that average osteoclast lifespan is not determined *a priori*, but depends instead on the size of the region where bone remodeling has to be performed. In this manner, the larger the size of the region to be remodeled, the longer the lifespan of osteoclasts called to remove old bone there. Moreover, simulation of the model reveals that such variable turnout in osteoclast number remains finely tuned with the number of other cell types involved, so that the whole process remains tightly regulated. This fact is illustrated in Fig 7 below, where mean OCL lifespan is plotted in terms of BRC depth.

### The BMU software is robust to signal noise

**Fig 8:**
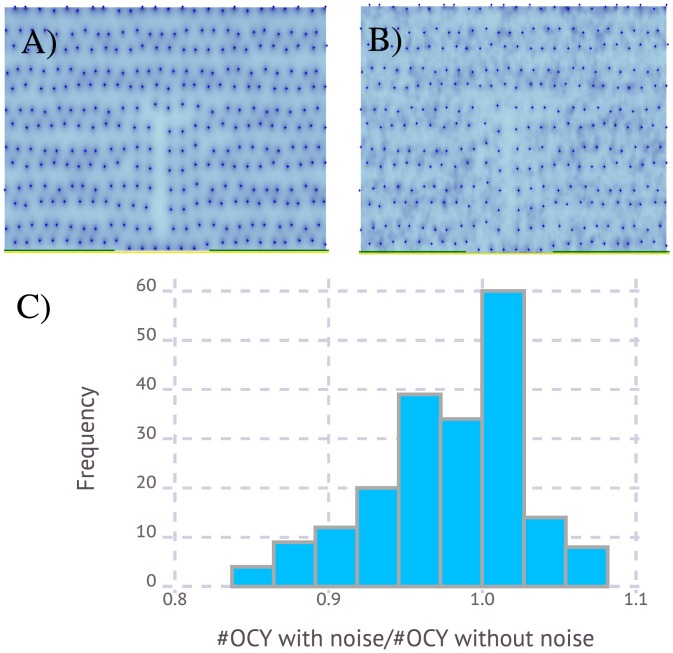
Effect of signal noise in BMU operation. **A)** Simulation corresponding to a bone remodeling without noise in signal S diffusion. The picture corresponds to the new bone after remodeling. **B)** In spite of noise in signal S, the new distribution of OCY is similar to A. **C)** Histogram of the ratio between the number of newly formed OCY in the BRC after bone remodeling with and without noise (the result was obtained after 200 simulations of the model).

As we have seen above, cell dynamics within a BMU depends on cell-to-cell communication mediated by signals that diffuse through the bone matrix. The coupling of old bone resorption and new bone formation can therefore be affected by signal noise due, for instance, to bone matrix spatial heterogeneity. In Fig 8 we show that the suggested BMU software is robust to environmental noise.

## Discussion

In this work we have proposed and analysed a minimal model for bone remodeling as carried out by Bone Multicellular Units (BMUs) to replace old bone with new, in a process that is frequently triggered by changes in mechanical load. We have already recalled that such remodeling occurs throughout the entire human lifespan [14]. BMUs activity is usually carried out on small regions, of about 4000 microns in width [9], and therefore the total number of cells involved in each remodeling event is comparatively small. The previous length scale can also be used to characterize micro-fractures (arising for instance from physical exercise) which are also self-repaired in the same manner. BMU operation can thus be viewed as the basic building step to ensure homeostasis in bone tissue. When normal function in bone is compromised by large-scale hazards (such as, fractures), more sophisticated repair mechanisms, particularly those involving inflammatory signals, blood clot formation and the production of different callus templates for bone repair are involved. These mechanisms have not been discussed here.

Our goal in this work was to identify, by means of a mathematical model, a simple algorithm for BMU operation. To do this, we have kept to a few basic principles. First, at any time during that process, each cell among the different lineages present in a BMU should individually select one from a restricted set of actions: divide, die, migrate or differentiate. Such choice i assumed to be non-random, but rather determined by feedback from the surrounding medium into the internal dynamics of molecules which act as decision inhibitors. No previous planning is presupposed, genetically nor otherwise. Second, only a minimal number of signals are selected that allow for BMU functioning. As indicated in a previous Section, molecular cues acting as those retained in our model have been already described. In particular, various cell decision inhibitors have been identified, and effects induced by the signals retained have been shown to be caused by chemical mediators experimentally detected. However, our work was not aimed at producing a comprehensive model where all data currently known could be fitted. We have instead attempted to show what the basic ingredients of an operational BMU could be like. Implicit in this approach is the assumption that some signals already identified are multi-functional (thus yielding different effects in different cell lines) and that, in general, some signaling networks observed may be redundant, perhaps created via different paths in the course of convergent evolution. Hence, we do not claim that our proposal is the only possible one. In principle, there could be alternative minimal models for what we have called a BMU software. We believe however, that the degree of complexity retained in our model could not be significantly reduced by alternative mechanisms.

Selecting a minimal model has the advantage of simplifying with a comparatively small number of parameters. Concerning this last issue, we wish to emphasize that no parameter-fitting attempt has been made here. Our concern was to show that the model proposed has not only the potential to reproduce standard features in bone remodeling, but also to draw conclusions about the manner in which this process occurs that could not be anticipated *a priori*, before simulating it. Accordingly, we are content to select a set of coefficients for which such goals are achieved. Parameters appearing in the actual BMU software should be of a structural nature, and their precise values have to be experimentally determined. In fact, the circuitry underlying BMU operation is expected to be rather sensitive to small variations in such parameter values, a fact repeatedly observed in processes that have emerged in the course of evolution. An example of such situation is provided by the Krebs cycle [50, 51] which consists of a network of biochemical reactions taking place at very precise and proportional rates. On the other hand, it is well known that a given phenomenon can be fitted by utterly distinct models, relying on different principles, upon appropriate choices of a suitable (and often rather modest) number of parameters specified in them [52, 53]. We have shown herein that the parameters retained in our model can be selected so that BMU standard operation is reproducible and, importantly, consequences such as the dependence of osteoclast lifespan on the size of the region to be repaired can be inferred, even though it may not have been clear before the model was simulated. We do not claim, however, that the results obtained provide a validation of the model. In our opinion, such validation could only derive from experimental determination of such values.

We conclude this discussion by briefly remarking on possible future directions arising from this work. To begin with, the experimental question of determining the operational parameters introduced in our model should be stressed once again. When this is done, we would have in place a BMU chip structure to be used as a building block for operational purposes. One could then simulate on models such as that presented here the impact of external cues in the modulation of the repair process, either to slow it down or to accelerate it. Finally, as the size of the region to be repaired increases, a threshold should be reached beyond which inflammatory signals become relevant, and a new and more complex stage is entered. A consolidation of these two cases could be instrumental in designing techniques to foster bone fracture repair under more extreme circumstances. We intend to pursue some of these enhanced possibilities.

## Supporting information

S1 Appendix Details of computer simulations of the model of BMU operation during bone remodeling. (PDF)

S1 Movie One sample of the simulation (from initial OCY apoptosis to final equilibrium and renewed bone). (PDF)
