# Supplementary material for: Bone remodeling: A tissue-level process emerging from cell-level molecular algorithms
Source: PLoS One. 2018 Sep 19;13(9):e0204171. doi: 10.1371/journal.pone.0204171 (PMC6145577; doi:10.1371/journal.pone.0204171)
Supplement: S1 Appendix — (PDF) [file pone.0204171.s001.pdf]

Details of computer simulations of the model of BMU operation during bone remodeling

Time evolution of the BMU

- **Initialization**: Simulations take place in a two-dimensional cellular automata (CA) of 150 x 250 boxes. 125 rows of this matrix are initially occupied by bone matrix and the rest by bone marrow. A row of OBL lies in the interface between them. OCYs are initially located at fixed, regularly spaced positions within the bone matrix. A random perturbation is then applied to their positions to avoid any potential effect of symmetries on the simulations of the model. At this point, OCYs and OBLs release signals that diffuse through the bone matrix until an equilibrium is attained. This equilibrium is determined by the balance between signal production and decay. Periodic boundary conditions are considered on both lateral sides of the region considered, and zero signal flux is imposed on the top and the bottom. A region of OCY apoptosis is randomly defined within *a priori* defined boundaries ($Depth :=$ maximum depth and $Width :=$ maximum width).

- **Time evolution**:

  Simulations of the model occur in discrete time steps of length $\Delta t = 0.01$. The input for an iteration of the model corresponding to time $t = t_0$ includes a set of vectors (one for each of the cells coexisting in the model at that time) with the coordinates of the box occupied by the cell, and its state variables (amount of inhibitory molecules).

  1.- OBL

  The state variable considered in the model for the i-th OBL is the amount of activation inhibitor d_{B_i}. The corresponding vector is:

  $$v_{B_i}(t_0) = \{(x, y), d_{B_i}(t_0)\}$$

  2.- OBA

  For each OBA there are three different inhibitory molecules, namely c_A, d_A and a_A, blocking cell division, differentiation and apoptosis. The state vector for the i-th OBA is:

$$v_{A_i}(t_0) = \{(x,y),(c_{A_i}(t_0), d_{A_i}(t_0), a_{A_i}(t_0))\}$$

3.- OCP

Differentiation of OCP into OCL is controlled by the inhibitor d_P, so that the state vector of the i-th OCP is given by:

$$v_{P_i}(t_0) = \{(x,y), d_{P_i}(t_0)\}$$

4.- OCL

Active osteoclasts dig into old bone while alive before they die by apoptosis. The state vector for the i-th OCL includes the amount of apoptosis inhibitor a_{C_i}:

$$v_{C_i} = \{(x,y), a_{C_i}(t_0)\}$$

Input also includes the values of bone density b and signals S, T and R at each box of the CA. The implementation was carried out with two different tools. The Mathematica platform (Wolfram Research, Inc., Mathematica, Version 9.0, Champaign, IL) was used to obtain Figure **??**. The remaining figures and simulations were carried out with the open source *Julia Language* [1], which is endowed with internal mechanisms as *multiple dispatch* and JIT compilation that allowed us to significantly reduce the computational time.

Each iteration starts with the update of cell internal inhibitors according to the equations that describe the dynamics of the state variables (see Models Section). If neither the amount of bone at a box occupied by a OCL or some of the inhibitors goes to zero during a time step, the model moves to the next time step. Alternatively, the model displays different behaviors depending on what variable (or variables) have gone to zero:

1.- OBL activation inhibitor (d_B)

This corresponds to the activation of one or more OBLs. In this case, the activated OBL are removed from the list of cells of the model, and their positions in the CA are occupied by newly formed OBAs.

2.- OBA division inhibitor (c_A)

OBAs form a growing front of cells that progresses by division behind the cutting cone created by OCLs. By construction, only OBAs that are not located in boxes adjacent to bone can divide. Moreover, OBA division can only occur if some of the neighboring positions is free. In this case, the position of a new OBA is selected at random from the free neighbors in the CA.

4.- OCP differentiation inhibitor (d_P)

OCPs are located adjacent to the layer of OBLs. In case of activation, the corresponding OCP is replaced by a newly formed OCL.

5.- OCL apoptosis inhibitor (a_C)

OCLs whose apoptosis inhibitor disappears are removed from the set of cells in the model for the next iteration.

6.- Bone removal by OCPs (b_P)

OCLs remove old bone from the box where they are located according to equation (7). When the amount of bone goes to zero, the OCL moves to one of the unoccupied adjacent boxes in the bone matrix. The OCL chooses the closest box that is farther from the OBL layer. OBAs deposit osteoid matrix at a fixed rate in positions attached to bone, and where bone was previously removed from the box. After cell fate choices, the set of cells is updated. Cell produce signals which subsequently diffuse and decay. The model starts a new iteration.

- **End of the simulation**:

  Simulations end when the bone remodeling process is completed (old bone has been replaced by new osteoid matrix) and a new equilibrium (for signals S and T) has been reached. A video showing one sample of the simulation (from initial OCY apoptosis to final equilibrium and renewed bone) is provided in file **S2_Movie**. The movie should be played with Adobe Acrobat Reader®. A list of parameter values used in the simulation of our model is provided in Table A1.

Pseudo-code guidelines

Input parameters

t_{max} := maximum duration of simulations

$Depth$:= maximum depth of the apoptosis region

$Width$:= maximum width of the apoptosis area

$bone(x, y)$:= 1 if the box is occupied by bone at initial time and 0 otherwise

$position(OCY, 0)$:= initial position of OCYs

$position(OBL, 0)$:= initial position of OBLs

$position(OCP, 0)$:= initial position of OCPs

Structural parameters

α_k^h := effect of external signals of type h on the dynamics of inhibitors in cells of type k

$D_B :=$ maximum amount of inhibitor d_B that can accumulate inside a OBL

$dp_h(d) :=$ diffusion profile of signal h as a function of distance d

$\gamma_h :=$ rate of decay of signal of type h

Variable parameters

$\{a_{k0}, d_{k0}, c_{k0}\} :=$ amount of inhibitors in newly formed cells of type k

δ_A Distance of OCY inhibition of OBA differentiation

$Q_k^h :=$ rate of production of signal of type h by cells of type k

Initialization

1 Input the bone matrix $bone(x, y)$

3 Input the the locations of OCY, OBL and OCL.

4 Production, diffusion and decay of signals S and T until equilibrium

5 Define a region of OCY apoptosis

6 Set $t := 0$

7 **REPEAT**

  8 Input the set of positions and state variables of all cells

  9 Input the amount of signals S, T and R at each box of the CA

  10 Integration of the equations for the internal inhibitors for a time step Δt

  11 If $(d_{Bi}(x, y; t) \leq 0)$ then remove the i-th OBL and add a new OBA at box (x, y)

  12 If $(c_{Ai}(x, y; t) \leq 0)$ then

    13 add a new OBA at a box adjacent to (x, y)

    14 recalculate OBAs positions

  15 If $(a_{Ai}(x, y; t) \leq 0)$ then

    16 remove the i-th OBA

    17 recalculate OBAs positions

  16 If $(d_{Ai}(x, y; t) \leq 0)$ then

    17 remove the i-th OBA

    18 add a new OCY at (x, y)

    19 recalculate OBAs positions

  20 If $d_{Pi}(x, y; t) \leq 0$ then

    21 remove the i-th OCP

    22 add a new OCL at (x, y)

23 If $a_{Ci}(x, y; t) \leq 0$ then remove the i-th OCL

24 If $b_{Bi}(x, y; t) \leq 0$ then

  25 set $bone(x, y) = 0$

  26 move the OCL to a new position in the bone matrix

27 Cell production, diffusion and decay of signals S, T and R during the time step Δt

13 set $t = t + \Delta t$

  # We remark that several cell decisions can take place simultaneously

32 **UNTIL** the end of bone remodeling process

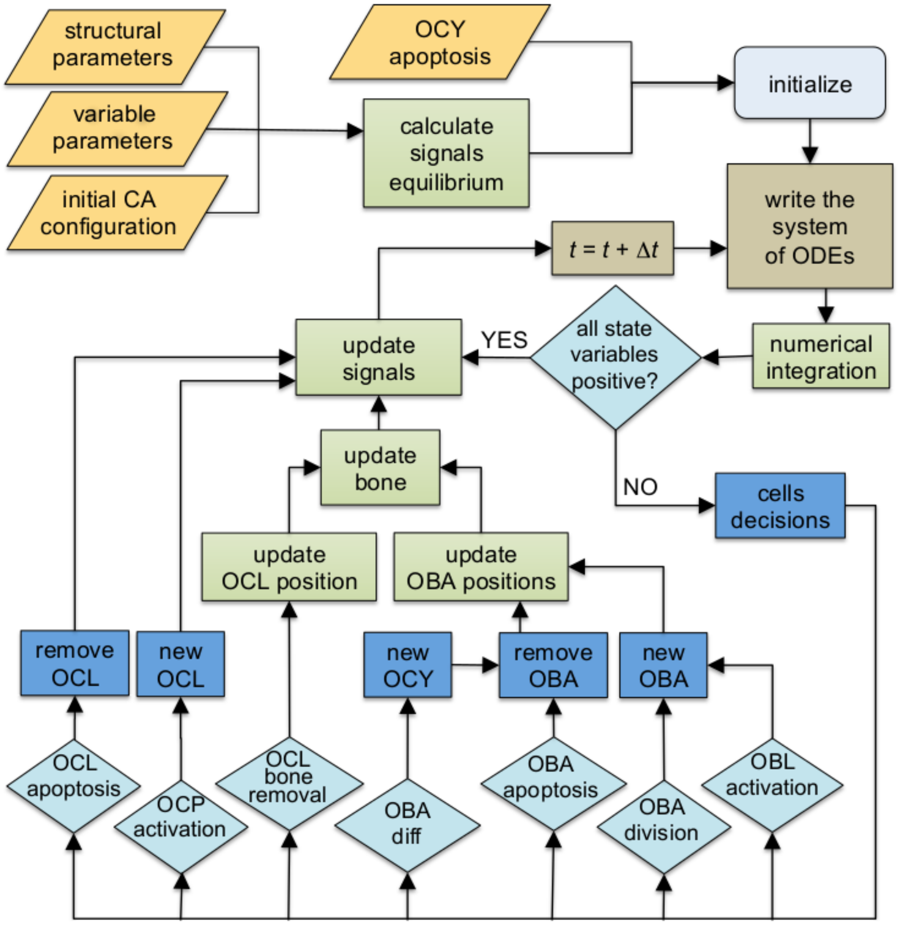

Figure A.1: **Flowchart of the proposed model of BMU software.**

Parameter	Description	Value
(Depth, Width)	Maximum depth and widht of the OCY apoptosis region	(80, 80)
d_{B0}	Initial amount of differentiation inhibitor in newly formed OBLs	1
D_B	Maximum amount of differentiation inhibitor in OBLs	2
$(\alpha_B^{S_1}, \alpha_B^{S_1})$	OBL structural parameters	(2, 1)
(c_{A0}, a_{A0}, d_{A0})	Initial amount of inhibitors in newly formed OBA	(1, 1, 1)
$(\alpha_A^T, \alpha_A^{S_1}, \alpha_A^{S_2}, \alpha_A^{S_3}, \alpha_A^O)$	OBA structural parameters	(0.1, 0.5, 1, 1,0.2)
δ_A	Radio of OCY ihibition of OBA differentiation	9
d_{P0}	Initial amount of differentiation inhibitor in OCP	1
(α_P^R, α_P^S)	OCP structural parameters	(1, 5)
a_{C0}	Initial amount of apoptosis inhibitor in OCL	1
$(\alpha_C^{S_1}, \alpha_C^{S_2}, \alpha_C^{S_3}, \alpha_C^{S_4})$	OCL structural parameters	(3, 1, 3, 4)
$(dp^S(d), dp^T(d), dp^R(d))$	Diffusion profile of signals S, T and R	$dp^S(d) = dp^T(d) =$ $= dp^R(d) = Max(0, 4 - d)$
$(\gamma_S, \gamma_T, \gamma_R)$	Decay rates of signals S, T and R	(0.05, 0.05, 0.05)
(Q_Y^S, Q_Y^T)	Rates of production of signals S and T by OCYs	(10, 1)
Q_B^T	Rate of production of signal T by OBL	2
(Q_A^T, Q_A^R)	Rates of production of signals T and R by OBAs	(1, 10)

Table A.1: **Values of the parameters used in the numerical simulations of the model.**

References

[1] Julia: A Fresh Approach to Numerical Computing. Jeff Bezanson, Alan Edelman, Stefan Karpinski and Viral B. Shah (2017) SIAM Review, 59: 65-98.

doi: 10.1137/141000671. url: http://julialang.org/publications/julia-fresh-approach-BEKS.pdf.
