# Supplementary material for: Bone remodeling: A tissue-level process emerging from cell-level molecular algorithms
Source: PLoS One. 2018 Sep 19;13(9):e0204171. doi: 10.1371/journal.pone.0204171 (PMC6145577; doi:10.1371/journal.pone.0204171)

time 0.05

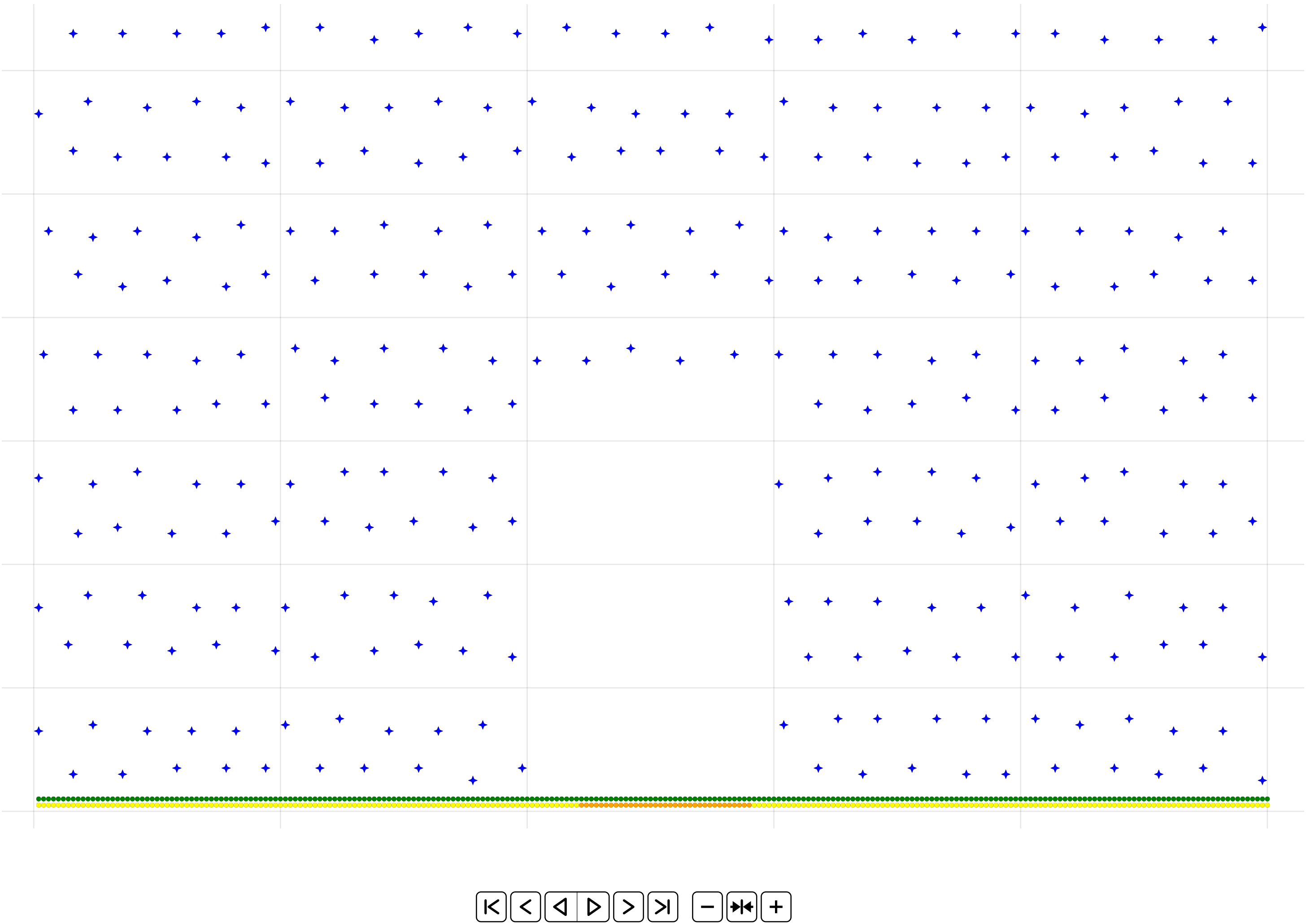

Supplement: S1 Movie — (PDF) [file pone.0204171.s002.pdf]
